# A health belief model-based community health education on mammography screening among reproductive-aged women in Ethiopia: a randomized controlled trial

**DOI:** 10.3389/fpubh.2024.1377173

**Published:** 2024-09-26

**Authors:** Feleke Doyore Agide, Gholamreza Garmaroudi, Roya Sadeghi, Elham Shakibazadeh, Mehdi Yaseri, Zewdie Birhanu Koricha

**Affiliations:** ^1^Department of Health Education and Promotion, School of Public Health, Tehran University of Medical Sciences, Tehran, Iran; ^2^School of Public Health, College of Medicine and Health Sciences, Wachemo University, Hossana, Ethiopia; ^3^Department of Epidemiology and Biostatistics, School of Public Health, Tehran University of Medical Sciences, Tehran, Iran; ^4^Department of Health, Behavior and Society, Institute of Health Sciences, Jimma University, Jimma, Ethiopia

**Keywords:** randomized trial, health education, mammography screening, health belief model, intervention

## Abstract

**Background:**

Early intervention in mammography use prevents breast cancer-related deaths. Therefore, this study aimed to apply health education interventions to mammography use in reproductive-aged women.

**Methods:**

This was a sequential exploratory design using qualitative and quantitative methods. The qualitative part used to gain insights into the design and development of interventions. For the randomized trial, a sample of 405 participants was recruited in each arm. The mean difference of interventions on the study variables was determined using a general linear model for repeated measures (ANOVA). For dichotomous variables, nonparametric tests (Cochran *Q*) were used. Path analysis was used to observe how the constructs of the Health Belief Model interacted. We registered PACTR database (https://pactr.samrc.ac.za/): “PACTR201802002902886.”

**Results:**

The study found that there was a strong interplay between perceptions of mammography screening and the intervention, showing that the likelihood of mammography use and comprehensive knowledge increased from baseline to endpoint (*p* < 0.005). Likewise, health motivation and all constructs of the health belief model had a statistically significant mean difference between the intervention and control groups (*p* < 0.005). However, the mean value of perceived barriers in the intervention group was statistically significantly reduced after three and six months (mean difference = −2.054 between Measure 1 and measure 2 and −1.942 between Measure 2 and Measure 3). The hypothesized causal paths effect of the model was explained by 64.3% that shows there is strong relationship of the variables significantly (*p* < 0.005).

**Conclusion:**

The study found that model-based mammography screening interventions had a significant impact at various time periods. We recommend future researchers consider the intensity and range of information to advance the field and figure out the problem while investigating the dose and peak of the intervention.

## Introduction

In developed countries, the decline in breast cancer mortality observed over the past three decades is partly due to intensive interventions and improved patient management, which may affect the benefit-to-harm ratio of mammography screening (1–3). According to global estimates, breast cancer affects approximately 2.1 million women annually and is the leading cause of cancer-related death among women in developing and developed countries (4, 5). Recent studies in the Western world showed an absolute reduction in breast cancer risk associated with cancer education about early detection and screening. The difference, even in the effectiveness of treatment and screening, is increasing (5). Although the prevalence of breast cancer is higher in the developed world, the rate in developing countries remains unacceptably high (5–9).

Breast cancer is the deadliest cancer in Ethiopia. Of course, early detection and self-referral for mammography screening have led to a noticeable change, recognizing that the timing of detection influences the effectiveness of breast cancer treatment (9–11). Many factors influencing the use of mammography could change as public health initiatives are introduced and poorly understood (11). For this reason, several observational studies have identified factors that lead to the occurrence of breast cancer (10–14). Therefore, intuitive scientists worldwide have suggested that the implementation of recommended breast cancer intervention methods, such as mammography, has a significant impact on early detection (15, 16). Breast cancer education has a significant impact on increasing awareness of early detection and improving chances of survival (15–17).

In Ethiopia, despite various breast cancer prevention mechanisms suggested by health professionals, early recognition of the symptoms and self-referral for treatment are still in question, and their chances of survival are nil due to a late report (8–10). Several observational studies have been conducted on mammography use among women, but none of these were interventional studies among women of reproductive age in Ethiopia. Regarding the art of mammography screening in Ethiopia, most of the screening services are given in the central part of the country, and a decade of collaborative work and lessons learned from developed countries to improve breast cancer outcomes. Though evidence shows poor awareness of breast cancer symptoms, prevention mechanisms, risk factors, and treatment options has usually been associated with patient delay in seeking help, the service availability to the have’s and have-not’s at the community level is limited, making treatment less effective and having a having a minimal survival rate (9–11). Thus, binding the community to seek the health services (mammography) where they are found and what the cost is (15, 16). Various health belief model-based studies predicted the perception of the individuals in one or another behavior (11, 12, 16, 17).

HBM is a socio-psychological model that attempts to explain and predict health behaviors in terms of certain belief patterns by focusing on the attitudes and beliefs of individuals. It was developed by social psychologists to explain the lack of public participation in health screening and prevention programs. Since then, it has been adapted to a variety of long-and short-term health behaviors, including breast screening behaviors. The HBM addresses the individual‘s perceptions of the threat posed by a health problem (susceptibility, severity), the benefits of avoiding the threat, and factors influencing the decision to act (barriers, cues to action, and self-efficacy); it also states specific health beliefs related to the health problem and recommended health actions that influence the likelihood of taking recommended health actions (mammography use) (18–21). Therefore, this study aimed to apply health education interventions to mammography use in women of childbearing age within the theoretical framework of the Health Belief Model (HBM). Moreover, the study hypothesized that a health belief model-based community health education on mammography screening among reproductive-aged women will bring amicable change in Ethiopia.

## Materials and methods

### Study design, populations and setting

This was a sequential exploratory design using qualitative and quantitative methods. An exploratory qualitative was used to get insight from women and health workers to design and development of intervention using focus group discussions (FGD) and in-depth interviews, respectively and published elsewhere (21). A sample of 405 participants in each arm was recruited for a randomized trial in the quantitative part and evaluated at baseline and three and six months after the educational intervention. Then, a randomized controlled trial proceeded by cross-sectional study lasting for six months was used to assess effectiveness of the health education intervention on mammography use among reproductive-aged women. The study included women in the childbearing age group (15–49) who were physically and mentally capable of giving informed written consent and able to follow the provided intervention without any assistance, as well as willing to provide their consent and data to the researcher admit. The exclusion criteria were participants who could not stay until the intervention was completed/participants who were mobile during the intervention period and participants who did not attend more than two sessions of the training were excluded from the study.

This study was conducted on women of childbearing age in the Hadiya zone of central Ethiopia region. There were 332 kebeles and kifleketemas in the zone, as well as 13 rural districts and seven city administrations. Hossana, the region’s capital, is located 230 kilometers from Addis Ababa, the capital of Ethiopia. It is estimated that 1,850,104 people live in the zone. The estimate of women of childbearing age (15–49) is 193,967. The total number of health facilities in the zone corresponds to the Kebele number and others. Health extension workers and community health agents play a crucial role in the prevention of communicable and non-communicable diseases. The study was conducted between April 2018 and May 2019.

### Recruitment

At the time of data collection, we included six districts from the zone. We then selected 30 kebeles from the selected districts and distributed them equally between intervention and control groups (i.e., 30 kebeles were divided into 15 intervention groups and 15 controls). Systematic sampling was used to select each participant from each kebele by summing all *K* values in the initial 23 households. To prevent contamination of information, random assignment was used to ensure that control and intervention sites were far apart. We assigned 405 participants to each arm and distributed them proportionally across each kebeles. Hence, the total number of study kebeles was 30 based on the WHO sampling recommendation (WHO 2008) (20).

Women who were potentially eligible for the study were selected for enrollment, and each woman was invited to participate in the study through a verbal invitation from the principal investigator and health extension workers of each selected kebele. If she agreed to participate, an appointment was arranged. After written informed consent, women were accepted as study participants, baseline data were collected, and participants were assigned to either the intervention or control group. After informed consent and baseline data collection, participants in the randomized controlled trial were randomly assigned to one of two arms: intervention or control. The different kebeles were coded alphabetically (A, B, C, D, etc.) and the participants assigned to each kebele were given numerical codes (e.g., participants in kebele 1 were numbered 1,001, 1,002, 1,003, etc.). Following three months and six months, follow-up data was gathered from both groups. We confirm that the original protocol was prepared for all breast screening behaviors (breast self-examination, breast clinical exam, and mammography use as a sequential exploratory study including qualitative and quantitative, and the two baselines were published elsewhere (21, 22). The length of the data collection period exceeded the initial protocol’s stated duration. Interventionist, data collectors, statistician were not the same persons. Interventionists also acknowledged all those contacted in the intervention arm.

#### Sampling and sample size determination

In this randomized trial, a double population proportion formula was used to calculate the sample size. This included 77.6% of participants who had knowledge of breast cancer screening methods (P1 = 77.6%) (23); P2 is the prevalence of screening rates in the intervention districts (87.6%). (Assumption: increase of 10%); *K* is the coefficient of variation of the true proportions of the outcome variable across counties within each group; the margin of error is 5%, with a significance level of 5% (two-tailed), i.e., a 95% confidence interval of certainty. Since there is no study estimating *k*, the value is assumed to be 0.25. Then the sample size was 368. Finally, the sample size was further increased by 10% to account for contingencies such as non-responses or recording errors, i.e., 368 × 10/100 + 368 = 404.8 ≈ 405. Therefore, the final sample size was 810 due to the design effect.

#### Measurement and variables

The intended outcome of this study was the likelihood of mammography screening (perceived benefits minus perceived barriers). The exposure variables were socio-demographic factors, knowledge about breast cancer and mammography screening, and previous breast screening behaviors. Age, marital status, religion, place of residence, educational and professional status as well as the current living situation of the respondents are socio-demographic factors and measured by seven items. There are eleven knowledge questions with the answer format “yes” or “no.” If respondents did not know the correct answer, they were asked to mark the “I do not know” answer option instead of guessing. Respondents who answered 50% or more of all knowledge questions about breast cancer and mammography screening were considered knowledgeable.

Respondents who answered less than 50% of all knowledge questions about breast cancer and mammography screening methods were classified as not knowledgeable. Perceived susceptibility is the self-perception of a respondent’s vulnerability to breast cancer as measured by a total of five belief items on a 5-point Likert scale. The perceived severity of breast cancer is the respondent’s belief about the impacts of breast cancer severity, as measured by a total of eleven belief items on a 5-point Likert scale. Perceived benefits of screening are respondents’ beliefs about the effectiveness of the method as a breast cancer prevention strategy, measured by a total of five belief items on a 5-point Likert scale. The perceived barriers to breast screening are respondents’ beliefs about how simple it is to perform the particular preventative actions, measured by a total of ten belief items on a 5-point Likert scale. Self-efficacy is defined as a respondent’s confidence in using breast screening procedures on her own in any circumstance or setting to avoid breast cancer, as measured by a total of five belief items on a 5-point Likert scale. Cues to action are conditions in the respondents’ environment that may encourage people to adopt breast screening procedures using a yes/no response style and measured by a total of five items. Past behavior (practice) refers to reproductive-aged women’s exposure to mammography screening at least once throughout the recommended period to avoid breast cancer, as measured using nominal measurements and measured by a total of six items. Before generating a summed score for each concept, negative-worded items were reversed. Community-based health education intervention description: Health education intervention was prepared based on health belief model constructs which are interlinked with mammography screening behavior. On top of this, the intervention emerged out of qualitative parts that were taken as very important components to know salient beliefs in the study area and later used as a very important base for intervention designing. Participants in the intervention arm received community based educational intervention in every 15 days for 3 months and registered their names and phone numbers (even family phone numbers) for tracking and reminding purposes. Educational intervention was provided on mammography use by training and teaching using different methods and materials like poster.

All of the participants were promised of the confidentiality throughout the process. For this, the enumerated lists of the participants were secured from the registry book of health workers after getting the consent. Immediately, after baseline data collection, the participants were categorized as intervention and control groups. All the required information of the both groups were taken and then registered in a temporarily prepared attendance sheet and followed accordingly. The participants were given an appointment to a health center or health posts near to them where the usual community forum was being conducted or the usual community meeting places for their community forum. The interventionists together with health extension workers facilitate the condition and deliver health education.

The control group received the usual services from health extension workers. These participants only received a welcome message at the beginning to validate their entry into the study and a message at the end of the follow-up to thank them for their participation. However, at the end of the 6 months, the same education was provided for the controlled groups.

### Data management and analysis

**Figure 1:**
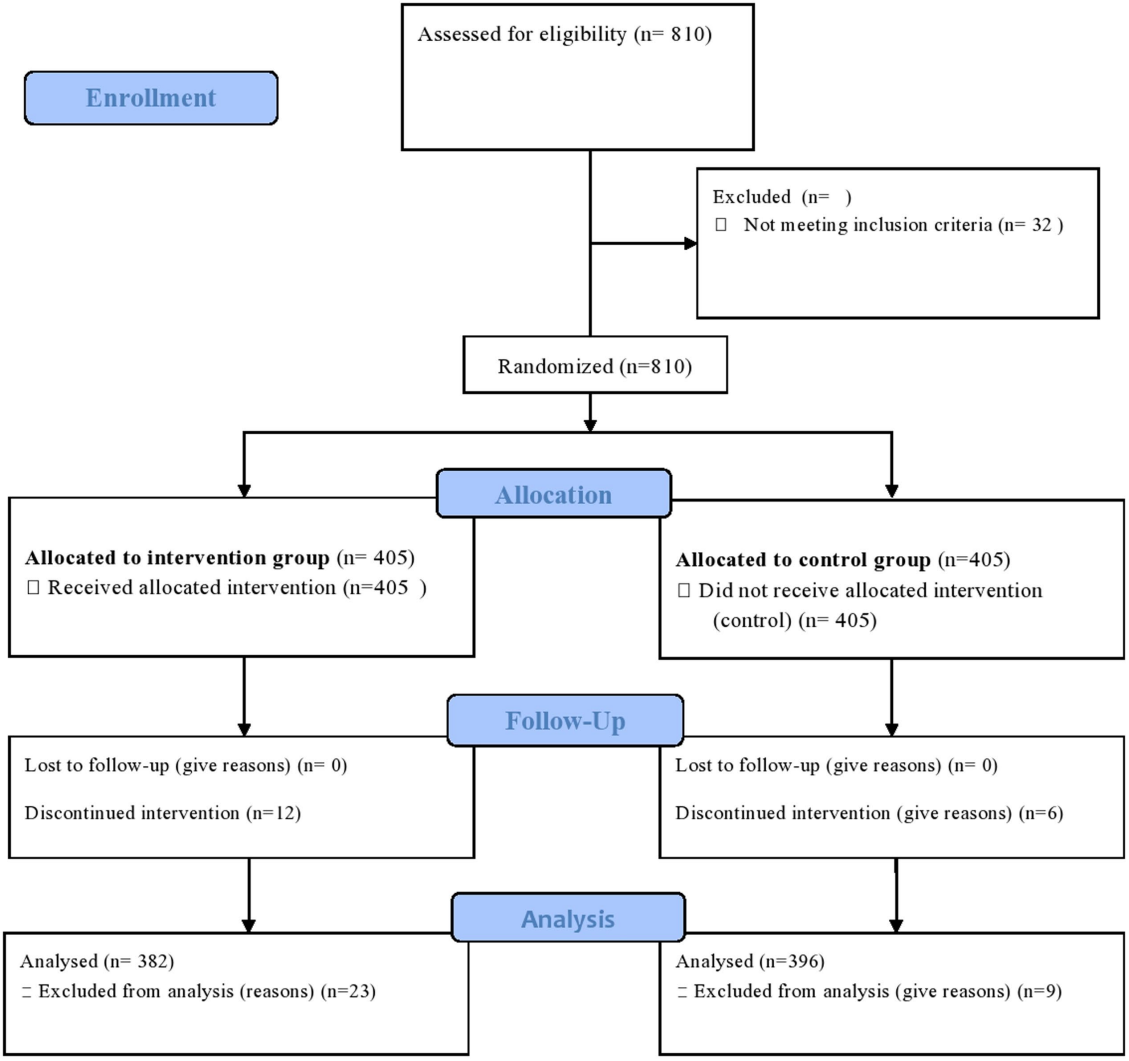
CONSORT flow diagram of the progress through the phases of a parallel randomized trial of two groups (that is, enrolment, intervention allocation, follow-up, and data analysis). Available at: http://www.consort-statement.org/consort-statement/flow-diagram.

Data were collected using designed and adapted structured interviewer-administered questionnaires. To ensure consistency, the questionnaires were translated into the local language and then back-translated into English by another person. There was a 2 day training session for data collectors and supervisors. Supervisors and principal investigators conducted direct supervision daily. Data were analyzed using SPSS V. 24.0. Before analysis, the data were checked for normality and homogeneity and then analyzed and interpreted by a research team and a biostatistician. Intervention results were analyzed according to the reporting standards of the Consolidated Reporting Standards for Trials (CONSORT standards) (Figure 1). To compare the intervention and control arms, the rate of mammography screening at baseline, three months, and the end of 6 months was compared using chi-square and ANOVA. A general linear regression model for repeated measures was used to determine the effectiveness of the intervention and predict independent predictors of mammography screening. And nonparametric tests (Cochran *Q*) were used for dichotomous variables to measure the effect size of mammography screening intervention. Path analysis was used to determine the direct and indirect effects of variables and to estimate the values of the coefficients in the underlying linear model at the end of 6 months.

### Ethics statement

The Research Ethical Review and Approval Board (RERB) of Tehran Medical University approved this for ethics (IR.TUMS.SPH.REC.1396.4088). Subsequently, the Research and Ethical Review Approval Committee (RERC: 6-19/5524) of the Southern Ethiopia Regional Health Bureau approved the study. This study also strictly adhered to the ethical guidelines of the Helsinki Declaration of Medical Research (24). Letters from TUMS-IC and the Southern Ethiopia Regional Health Office was given to the Hadiya Zone Health Department for legal permission. After the objectives and benefits of the study were explained in detail, each participant provided written informed consent. Study participants had the right to withdraw from the study at any time. Participants were also informed that their responses would remain confidential and their names would not be disclosed. To maintain ethical issues, the same training was offered to the controlled groups at the end of data collection. During the course of our investigation, we strictly adhered to all international and institutional ethical conventions for research on randomized control trials. This study was registered in the Pan African Clinical Trial Registry database (https://pactr.samrc.ac.za/) with the unique identification number PACTR201802002902886.

## Results

### Socio-demographic characteristics of the participants

At baseline, a total of 405 participants were assigned to each group as the intervention and control groups. However, a total of 778 women of childbearing age responded to the interview questionnaire throughout the study period, yielding a response rate of 96.05%. The mean age of participants in the intervention and control arms was 31.9 (SD 7.4) and 32.2 (SD 7.8) years, respectively. Thirty-two participants were excluded because they did not attend two sessions of mammography training. There were no statistically significant differences between the two groups for any socio-demographic characteristics at baseline (*p* > 0.05). However, after the intervention, there were significant differences in ethnic group, educational status, occupational status and living conditions (*p* < 0.05) (Table 1).

**Table 1:** Socio-demographic characteristics of the participants at baseline.

Variables	Category	Intervention and control categories		*p*-value
		Baseline		
		Intervention group (*n* = 405)	Control group (*n* = 405)	
		Number (%)	Number (%)	
Age	15–34	246 (60.7)	233 (57.5)	0.329
	35–49	159 (39.3)	172 (42.5)	
Current residence	Rural	205 (50.6)	320 (79.0)	0.427
	Urban	200 (49.4)	85 (21.0)	
Religion	Protestant	289 (71.4)	308 (76.0)	0.687
	Orthodox	70 (17.3)	71 (17.5)	
	Muslim	31 (7.7)	14 (3.5)	
	Catholic	15 (3.7)	12 (3.0)	
Marital status	Single	32 (7.9)	45 (11.1)	0.162
	Married	350 (86.4)	344 (84.9)	
	Divorced	23 (5.7)	16 (4.0)	
Educational status	Cannot read and write	265 (65.4)	188 (46.4)	0.0004*
	Can read and write	95 (23.5)	102 (25.2)	
	Primary school	11 (2.7)	30 (7.4)	
	High school	14 (3.5)	45 (11.1)	
	College and above	20 (4.9)	40 (9.9)	
Occupational status	House wife	282 (69.6)	285 (70.4)	0.045
	Employee	36 (8.9)	28 (6.9)	
	Merchant	28 (6.9)	39 (9.6)	
	Private business	33 (8.1)	32 (7.9)	
	Students	26 (6.4)	21 (5.2)	
Categorized income	<=1,500	366 (90.4)	377 (93.1)	0.339
	>1,500	39 (9.6)	28 (6.9)	

### Breast cancer knowledge and mammography screening

**Figure 2:**
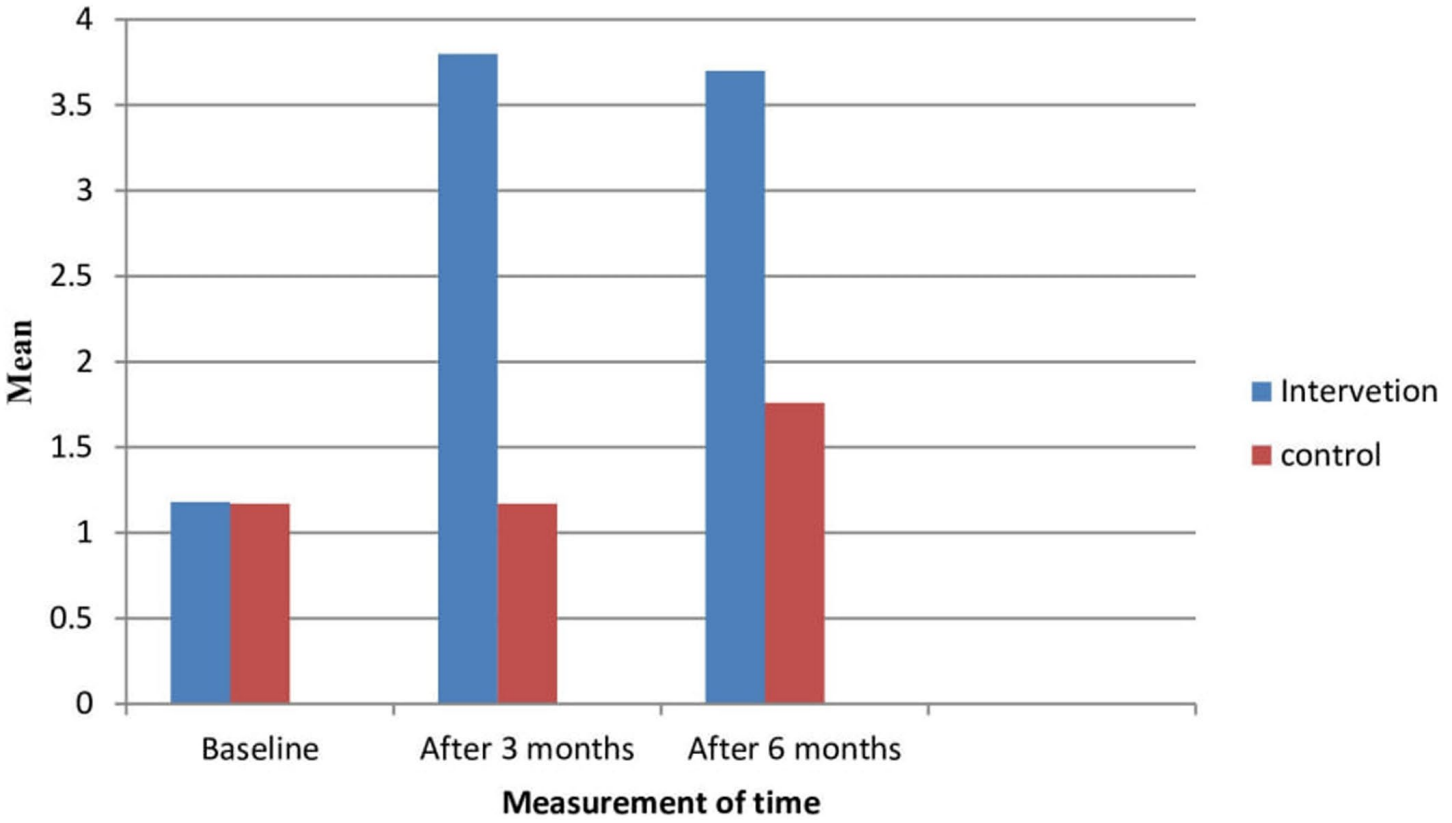
Knowledge of the participants about breast cancer and mammography screening in intervention and control groups.

At the baseline of the study, 95.6% of participants in the intervention and control groups had heard of breast cancer. However, at the baseline, 36.9% of the participants had already heard of the all breast screening methods including mammography screening. However, after the intervention, all participants in the intervention group had heard about mammography screening. However, there were no significant changes in prevalence in the control group. Similarly, participants’ mean comprehensive knowledge at baseline was 1.18 ± 0.54 and 1.17 ± 0.57 in both the intervention and control groups, with no significant difference. However, participants’ mean comprehensive knowledge increased by 3.8 ± 0.48 and 3.7 ± 0.53 after three months and six months of the intervention, respectively. However, in the control group, the mean (1.17 ± 0.57) increased after three months and at the end of the intervention, but there was no significant difference at both time points of data collection (1.76 ± 0.51 and 1.77 ± 0.52) (Figure 2).

### Perception towards breast cancer and mammography screening

The likelihood of mammography use of the participants was computed from the perception scores of the benefits minus barriers of the threat. Table 2 shows participants’ perceptions of breast cancer and the use of mammography. As a result, the likelihood of using mammography at baseline was 30.06% in the intervention group and 29.01% in the control group. However, the likelihood of using mammography at three months and at the end of the intervention at six months was 56.48 and 56.77%, respectively. At baseline, perceived susceptibility to breast cancer had mean values of (mean ± standard deviation) (16.9 ± 4.3) in the intervention group and (16.5 ± 4.6) in the control group based on threat appraisals. However, there was a significant mean difference after three and six months of intervention (*p* < 0.05). Likewise, the perceived severity of breast cancer at baseline had corresponding average values of (mean ± standard deviation) (38.1 ± 8.6) in the intervention group and the control group (37.2 ± 9.1). However, the significant difference was observed at three and six months (*p* < 0.05). The likelihood ratings, perceived benefits, and barriers of breast cancer screening methods had an average value of (mean ± standard deviation) in the intervention group (18.9 ± 3.6 and 37.4 ± 5.5) and in the control group (18.9 ± 3.5 and 35.8 ± 5.7) at the baseline, respectively. However, there was a statistically significant mean difference after three months of intervention and at the end of six months in the intervention group (21.4 ± 1.5 and 21.4 ± 1.6) in the respective order compared to the control group (*p* < 0.05) (Table 2).

**Table 2:** Mean scores of perception of the participants about breast cancer and mammography screening based on health belief model constructs.

Variables	Score ranges	Intervention and control categories across the study period							
		Baseline		Mid-line (After 3 months)			End-line (After 6 months)		
		Intervention	Control	Intervention	Control	*p*-value	Intervention	Control	*p*-value
		Mean ± SD	Mean ± SD	Mean ± SD	Mean ± SD		Mean ± SD	Mean ± SD	
Perceived benefits	5–25	18.9 ± 3.6	18.9 ± 3.5	21.4 ± 1.5	19.2 ± 4.6	0.000	21.3 ± 1.7	19.1 ± 4.5	0.00003
Perceived barrier	10–50	37.4 ± 5.5	35.8 ± 5.7	42.6 ± 3.2	35.2 ± 10.1	0.000	42.5 ± 4.1	35.7 ± 9.8	0.00001
Perceived Susceptibility	5–25	16.9 ± 4.3	16.5 ± 4.6	19.9 ± 2.1	16.4 ± 4.6	0.000	19.9 ± 1.9	17.0 ± 4.5	0.00001
Perceived severity	11–55	38.1 ± 8.6	37.2 ± 9.1	48.0 ± 3.8	41.5 ± 7.4	0.000	48.2 ± 2.2	41.8 ± 6.9	0.00011
Self-efficacy	5–25	18.1 ± 6.5	17.9 ± 7.5	21.4 ± 3.8	18.0 ± 7.9	0.000	21.4 ± 3.4	18.1 ± 9.3	0.00010
Cues to action	0–5	3.4 ± 2.2	3.7 ± 1.9	4.8 ± 1.5	3.9 ± 1.9	0.000	4.4 ± 1.8	3.8 ± 2.1	0.00014
Likelihood of mammography use	Weighted mean	30.06	29.01	56.48	31.31	0.000	56.77	31.21	0.00001

### Regression analysis to identify independent predictors of mammography screening

To examine the effect of interventions on the study variables, a general linear model of repeated measures was used. Table 3 shows a general linear regression model analysis of repeated measures comparing the mean difference (two-way ANOVA for repeated measures). As a result, there was a statistically significant mean difference between the intervention and control groups in the model constructs for health belief and health motivation (*p* < 0.005). Likewise, the intervention group’s mean perceived barrier score was statistically significantly lower after three and six months (mean difference = −2.054 between Time 1 and Time 2 and −1.942 between Time 2 and Time 3). However, the mean difference in action cues below one indicated that the intervention explained the least variance in the current context (Table 3).

**Table 3:** Mean difference of the perception scores to see the effect of the intervention using general regression model for repeated measure.

Variables	Measures (1, 2, 3)		Intervention vs Control (mean difference)	Standard error	95% Confidence interval for mean difference		*p*-value
					Lower bound	Upper bound	
Perceived susceptibility	Measure 1	Measure 2	1.359	0.198	0.879	1.840	0.00001
		Measure 3	1.328	0.220	0.856	1.834	0.00001
Perceived severity	Measure 1	Measure 2	6.152	0.262	5.282	9.221	0.00013
		Measure 3	6.316	0.265	5.431	9.182	0.00011
Perceived benefits	Measure 1	Measure 2	1.286	0.156	0.912	2.659	0.00001
		Measure 3	1.252	0.158	0.873	2.630	0.00001
Perceived barriers	Measure 1	Measure 2	−2.054	0.260	−2.570	−1.322	0.00010
		Measure 3	−1.942	0.268	−2.483	−1.196	0.00012
Perceived self-efficacy	Measure 1	Measure 2	2.721	0.176	2.299	4.890	0.00010
		Measure 3	2.723	0.173	2.342	4.754	0.00000
Cues to action score	Measure 1	Measure 2	0.453	0.087	0.236	0.667	0.00010
		Measure 3	0.437	0.084	0.325	0.685	0.00001
Health motivation	Measure 1	Measure 2	1.179	0.305	0.446	1.898	0.00001
		Measure 3	2.558	0.249	2.114	3.199	0.00012

Based on estimated marginal means; b. Adjustment for multiple comparisons: Bonferroni.

### Impact of health education on perceptions of each constructs (variance explained)

The variance of the impact of health education on perceptions of each construct was assessed and described in percentages. Table 4 shows the impact of each community health education intervention on each construct (variance explained by interventions). As a result, the community-based health education intervention accounted for 77.8% of the variance in knowledge, with a statistically significant effect on the intervention group (*p* < 0.05). In addition, the program had a statistically significant effect on health motivation, accounting for 41.4% of the variance (*p* = 0.000). Concerning threat appraisal, the intervention explained 20.8% of the variance in perceived susceptibility to and 23.5% of the variance in severity of breast cancer, with a statistically significant influence on the intervention group *p* < 0.05) (Table 4).

**Table 4:** General regression model analysis for repeated measures of mammography screening after adjustment for multiple comparisons (ANOVA) to see the effect of intervention.

Variables	Source	Df	*F*	Partial Eta squared	Sig.
Knowledge score	Intercept	1	34517.429	0.940	0.0000
	Intervention	1	3180.680	0.778	0.0001
Health motivation	Intercept	1	175552.614	0.957	0.0000
	Intervention	1	564.700	0.414	0.0000
Susceptibility	Intercept	1	45987.585	0.945	0.0000
	Intervention	1	206.951	0.208	0.0001
Severity	Intercept	1	73219.609	0.952	0.0000
	Intervention	1	241.420	0.235	0.0001
Benefit	Intercept	1	104699.566	0.954	0.0000
	Intervention	1	184.539	0.196	0.00011
Barrier	Intercept	1	141232.019	0.956	0.00011
	Intervention	1	682.320	0.459	0.00012
Self-efficacy	Intercept	1	69914.539	0.950	0.00003
	Intervention	1	250.449	0.242	0.00014
Cues to action	Intercept	1	10228.389	0.895	0.00011
	Intervention	1	8.041	0.010	0.00300

*Bonferroni tests of between-subjects effects of pairwise comparisons.

### Impact of health education interventions on actual behavior (effect size measurement)

Actual breast screening behavior was assessed as past behavior. This part included all the options of screening as a past history screening (breast self-examination, breast clinical exam and mammography use). Table 5 shows the effect size for dichotomous variables on screening behavior as determined by nonparametric testing (Cochran *Q*). Accordingly, the impacts of intervention in hearing about breast cancer were demonstrated, and breast screening method at various time periods or under varied conditions had a statistically significant influence on the study population (*p* < 0.05). The intervention had a statistically significant effect on breast screening perception (*p* < 0.05) and yielded greater percentages in perception than actual screening in case mammography screening. In terms of information source, participants’ exposure to media and health worker information rose considerably after intervention and was maintained in the maintenance stage (six months) (*p* < 0.05) (Table 5).

**Table 5:** Effect size measured for general breast screening using non-parametric tests (Cochran *Q*).

Variables	Categories	Measurement of time (breast screening behavior)							
		Baseline		At 3 months		At 6 months		Effect size	
		Intervention *n* (%)	Control *n* (%)	Intervention *n* (%)	Control *n* (%)	Intervention *n* (%)	Control *n* (%)	At 3 months *n* (%)	At 6 months *n* (%)
Ever heard BC?	Yes	383 (94.6)	391 (97.0)	393 (100.0)	394 (98.7)	382 (100.0)	391 (98.7)	22.5 (*p* = 0.000)	22.5 (*p* = 0.0001)
Heard screening methods?	Yes	161 (39.8)	138 (34.1)	393 (100.0)	142 (35.3)	382 (100.0)	140 (35.4)	140.1 (*p* = 0.000)	132.2 (*p* = 0.000)
Source of information	Health worker	157 (97.5)	119 (86.2)	393 (100.0)	124 (87.7)	382 (100.0)	122 (87.1)	95.0 (*p* = 0.025)	95.0 (*p* = 0.025)
	Media	99 (61.5)	84 (60.9)	177 (45.0)	72 (50.7)	173 (45.3)	71 (50.7)	62.2 (*p* = 0.000)	61.7 (*p* = 0.0001)
	Relative	99 (61.5)	84 (60.9)	139 (35.4)	91 (64.1)	135 (35.3)	89 (63.6)	75.1 (*p* = 0.000)	74.1 (*p* = 0.0001)
	Friends	81 (50.3)	59 (42.8)	167 (42.5)	68 (47.9)	164 (42.9)	67 (47.9)	0.7 (*p* = 0.413)	0.7 (*p* = 0.413)
BC screened	Yes	47 (29.2)	23 (17.2)	107 (27.2)	32 (22.5)	99 (25.9)	22 (15.7)	70.0 (*p* = 0.000)	70.0 (*p* = 0.0001)
Method of screening used?	Mammography	3 (6.4)	3 (13.0)	3 (2.8)	2 (6.3)	6 (10.6)	5 (21.0)	3.0 (*p* = 0.083)	1.0 (*p* = 0.317)
	BCE	12 (25.5)	5 (21.7)	9 (8.4)	5 (15.6)	9 (9.1)	5 (21.7)	3.0 (*p* = 0.083)	3.0 (*p* = 0.0831)
	BSE	32 (68.1)	15 (65.2)	95 (88.8)	25 (78.1)	90 (90.9)	18 (78.3)	73.0 (*p* = 0.000)	61.0 (*p* = 0.0001)
Frequency of breast screening	Sometimes	27 (57.4)	13 (56.5)	65 (60.7)	19 (59.4)	57 (57.6)	12 (52.2)	44.0 (*p* = 0.000)	29.0 (*p* = 0.0001)
	Usually	1 (2.1)	2 (8.7)	5 (4.7)	2 (6.3)	5 (5.1)	2 (8.7)	4.0 (*p* = 0.046)	4.0 (*p* = 0.046)
	Consistently	4 (8.5)	3 (13.0)	16 (15.0)	7 (21.9)	16 (16.2)	6 (26.1)	16.0 (*p* = 0.000)	15.0 (*p* = 0.0001)
	Others (once, ill)	15 (31.9)	5 (21.7)	21 (19.6)	4 (12.5)	21 (21.2)	3 (13.0)	5.0 (*p* = 0.025)	4.0 (*p* = 0.046)

### The effect of intervention across the study districts (Woredas)

**Figure 3:**
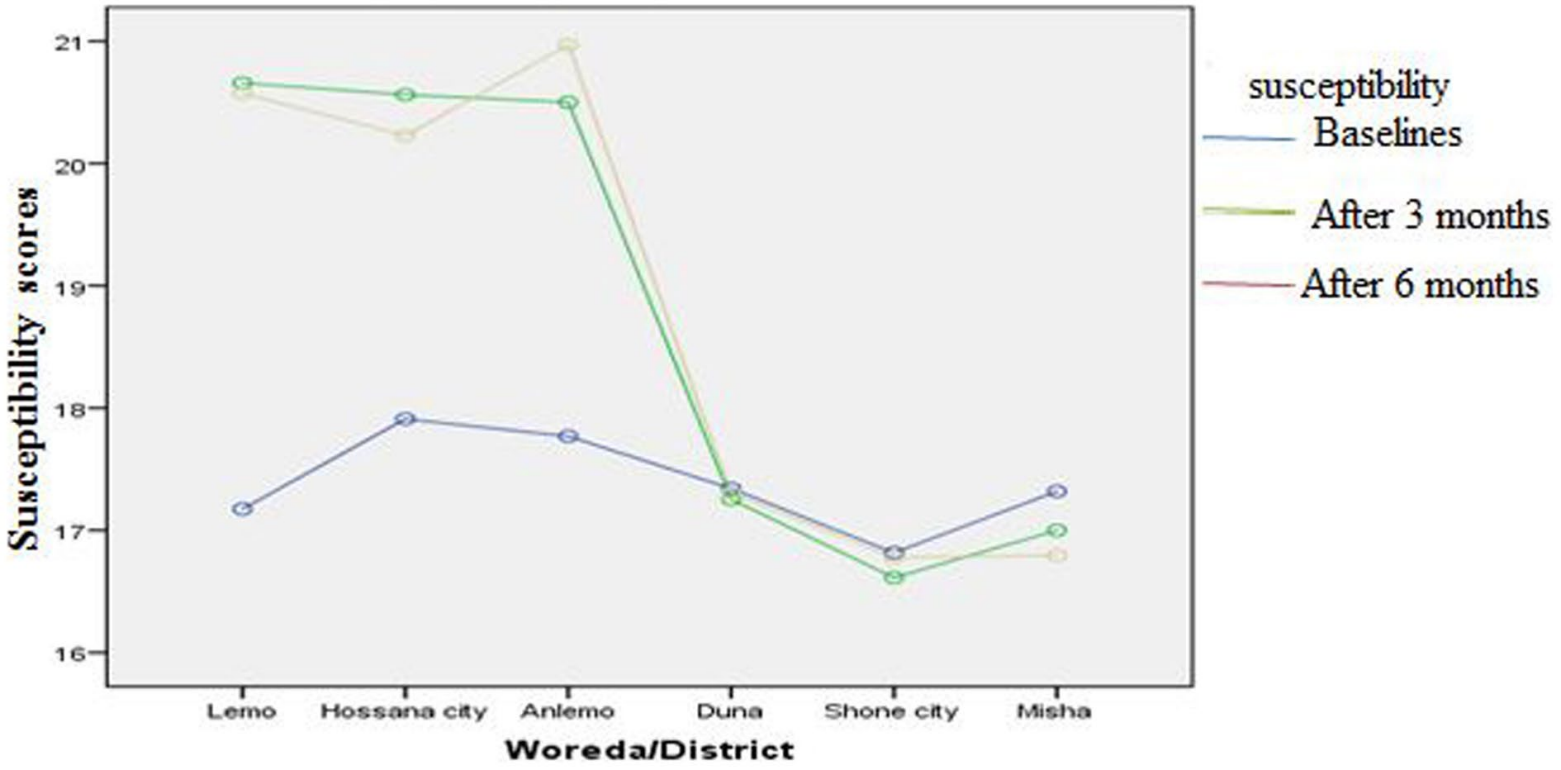
The effect of intervention on susceptibility of the participants across the study districts.

**Figure 4:**
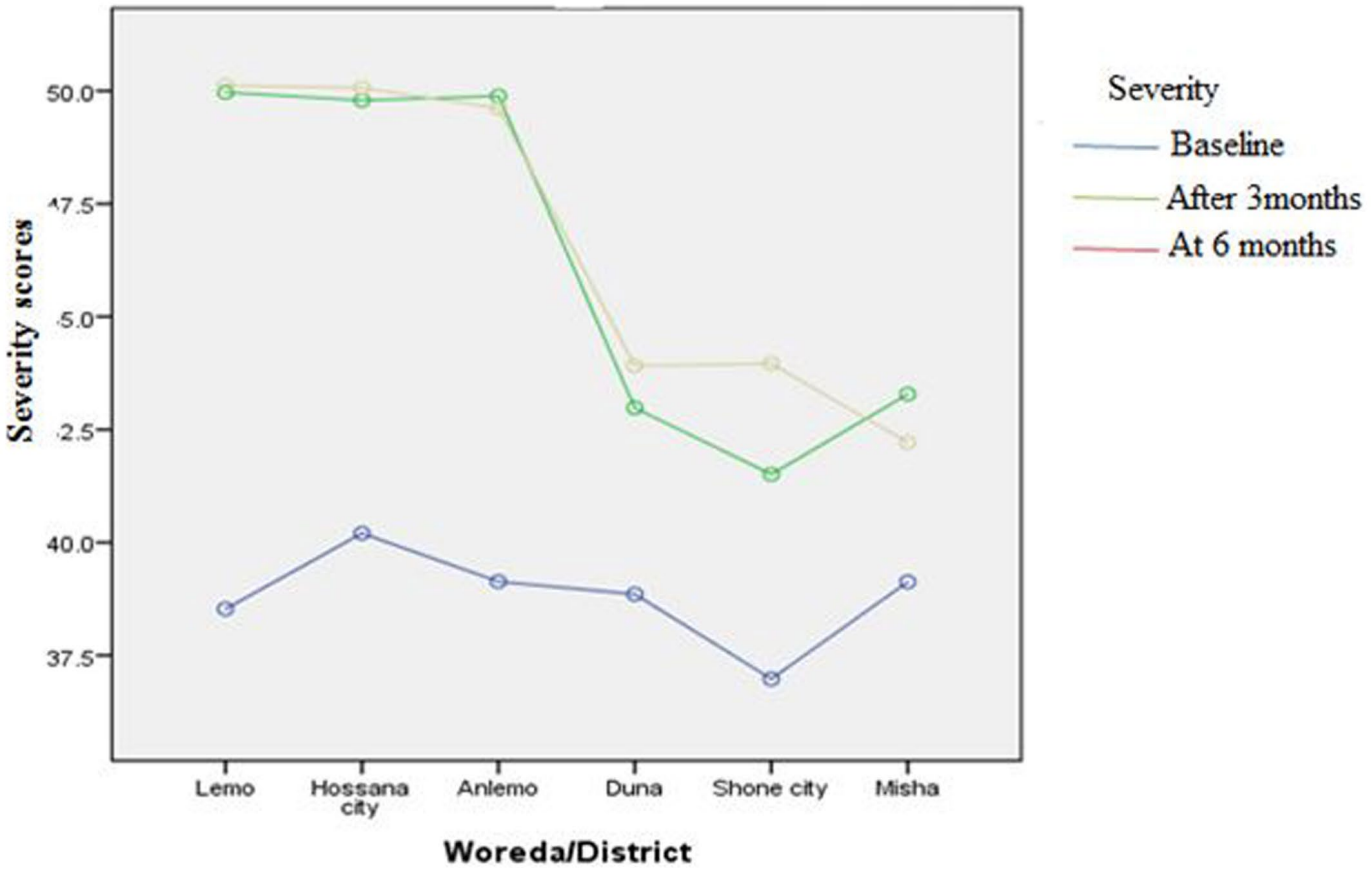
The effect of intervention on severity of the participants across the study districts.

**Figure 5:**
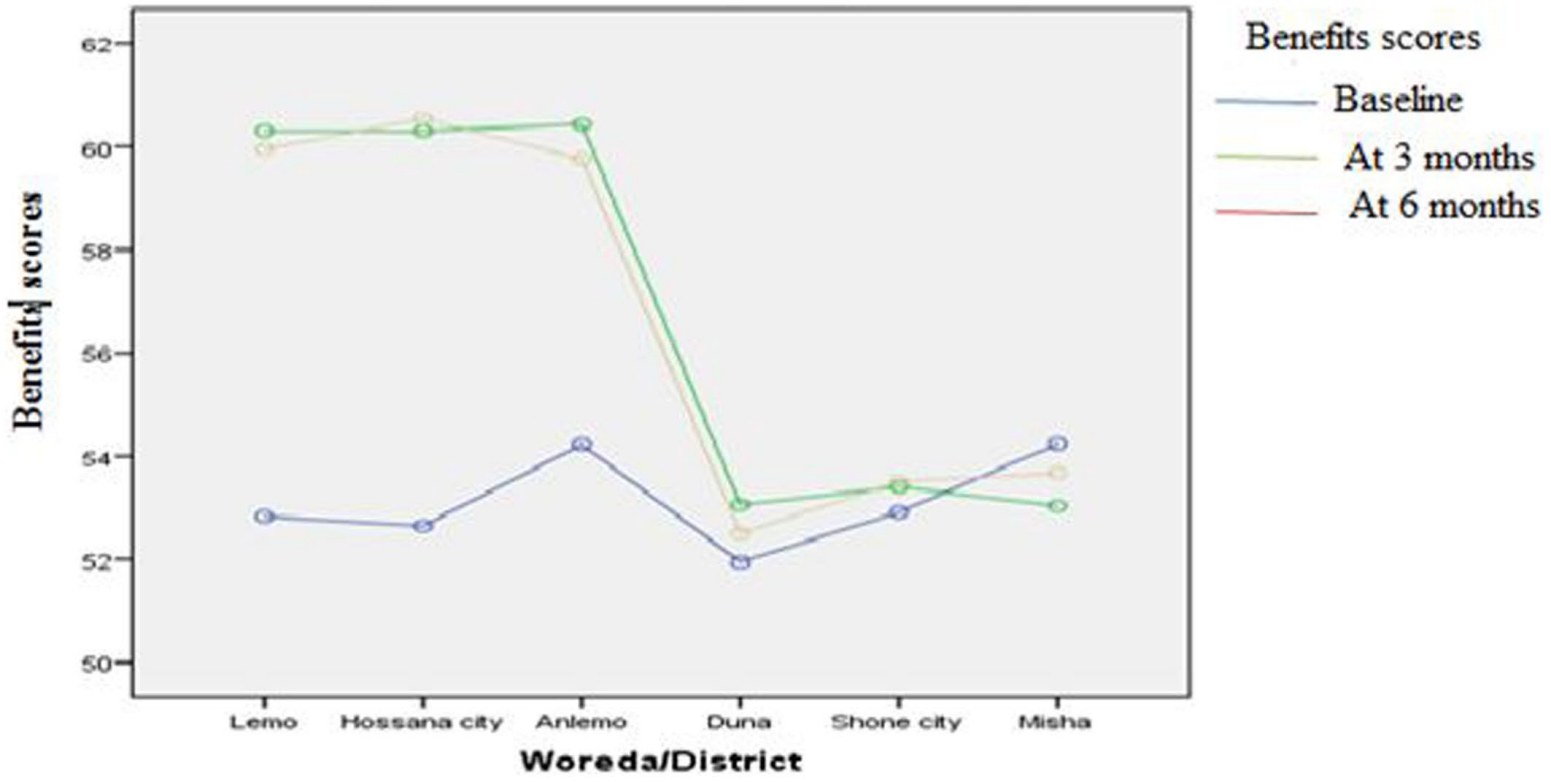
The effect of intervention on benefits of the participants across the study districts.

**Figure 6:**
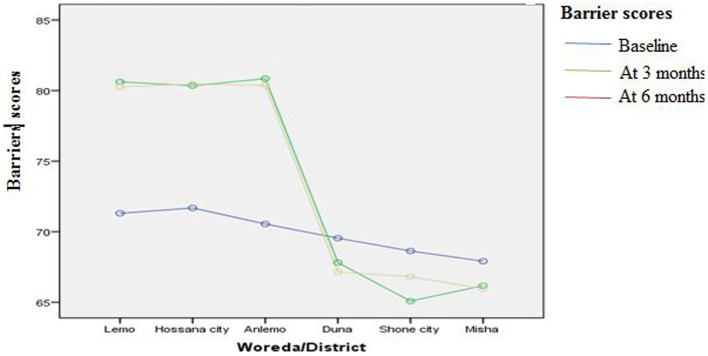
The effect of intervention on barriers of the participants across the study districts.

**Figure 7:**
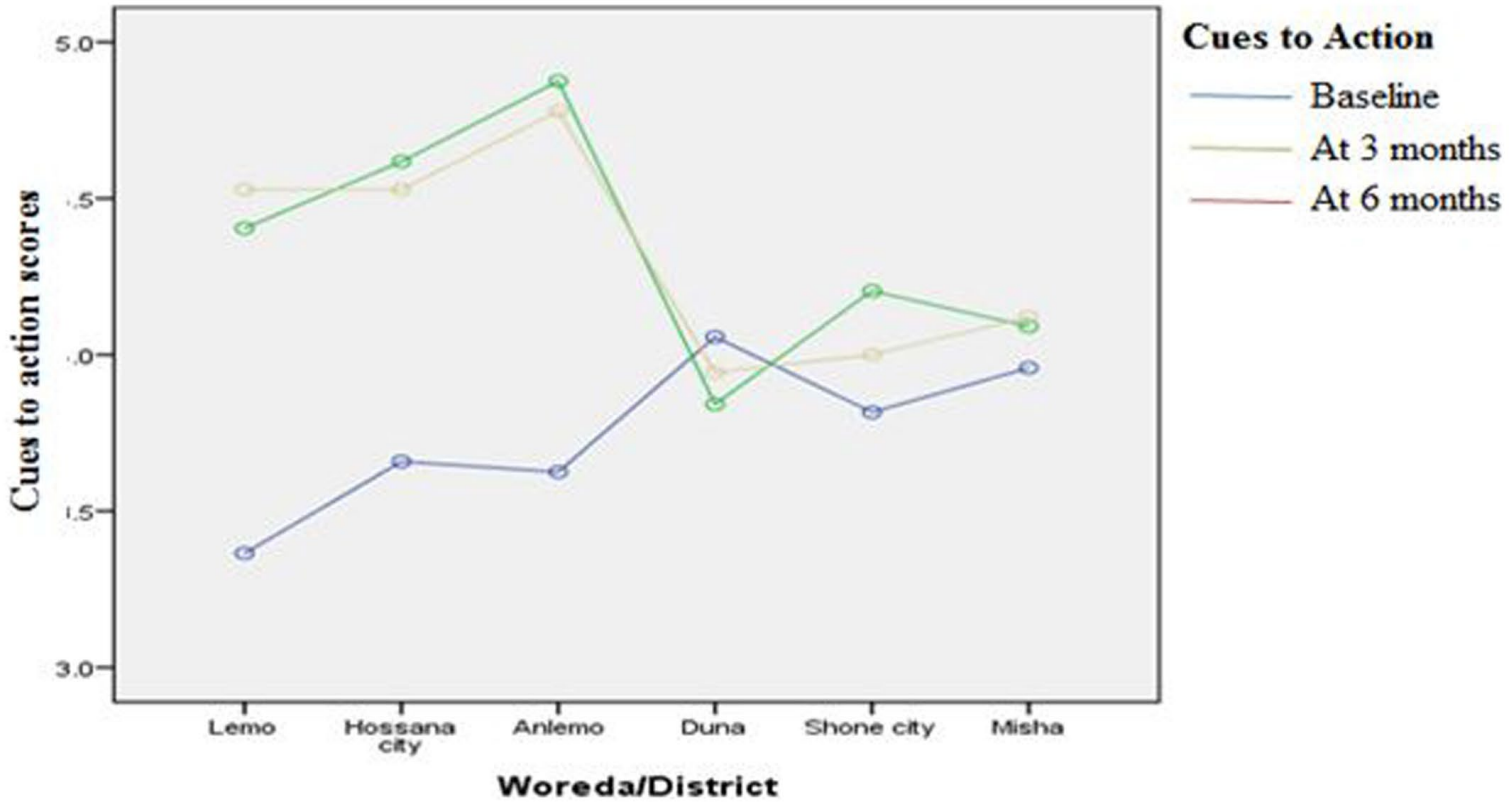
The effect of intervention on cues to action of the participants across the study districts.

**Figure 8:**
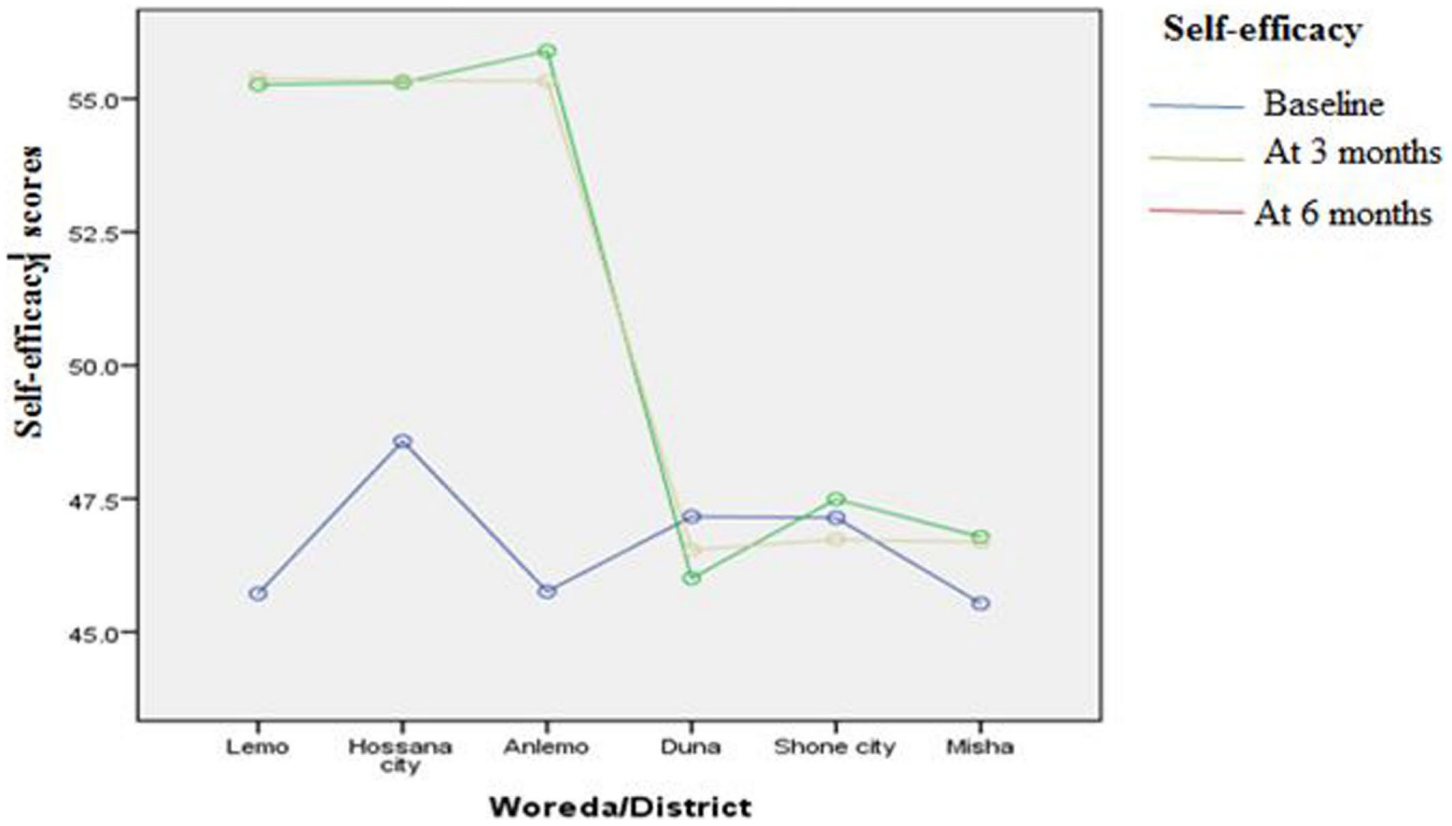
The effect of intervention on self-efficacy of the participants across the study districts.

Figures 3–8 presents the effects of interventions on outcomes across the study districts. This was analyzed by a general linear model for repeated measures (ANOVA) and regressive analysis obtained were depicted in the form of charts. Accordingly, generally, susceptibility, severity, benefits, self-efficacy, and cues to action scores were slightly the same across the intervention and control groups at baseline. Unlikely, the cues to action were significantly higher at baseline in Hossana town. However, after three and six months of intervention, the estimated regressive marginal means of measures indicated the intervention districts increased in all outcome variables. As far as the graphical presentation of the estimated mean is concerned, there were no visible differences across the groups at the maintenance stage (at six months). In each graph, the variation was fully described in three lines, starting from baseline to six months (Figures 3–8).

### The interactions of constructs of HBM on likelihood of mammography screening

**Figure 9:**
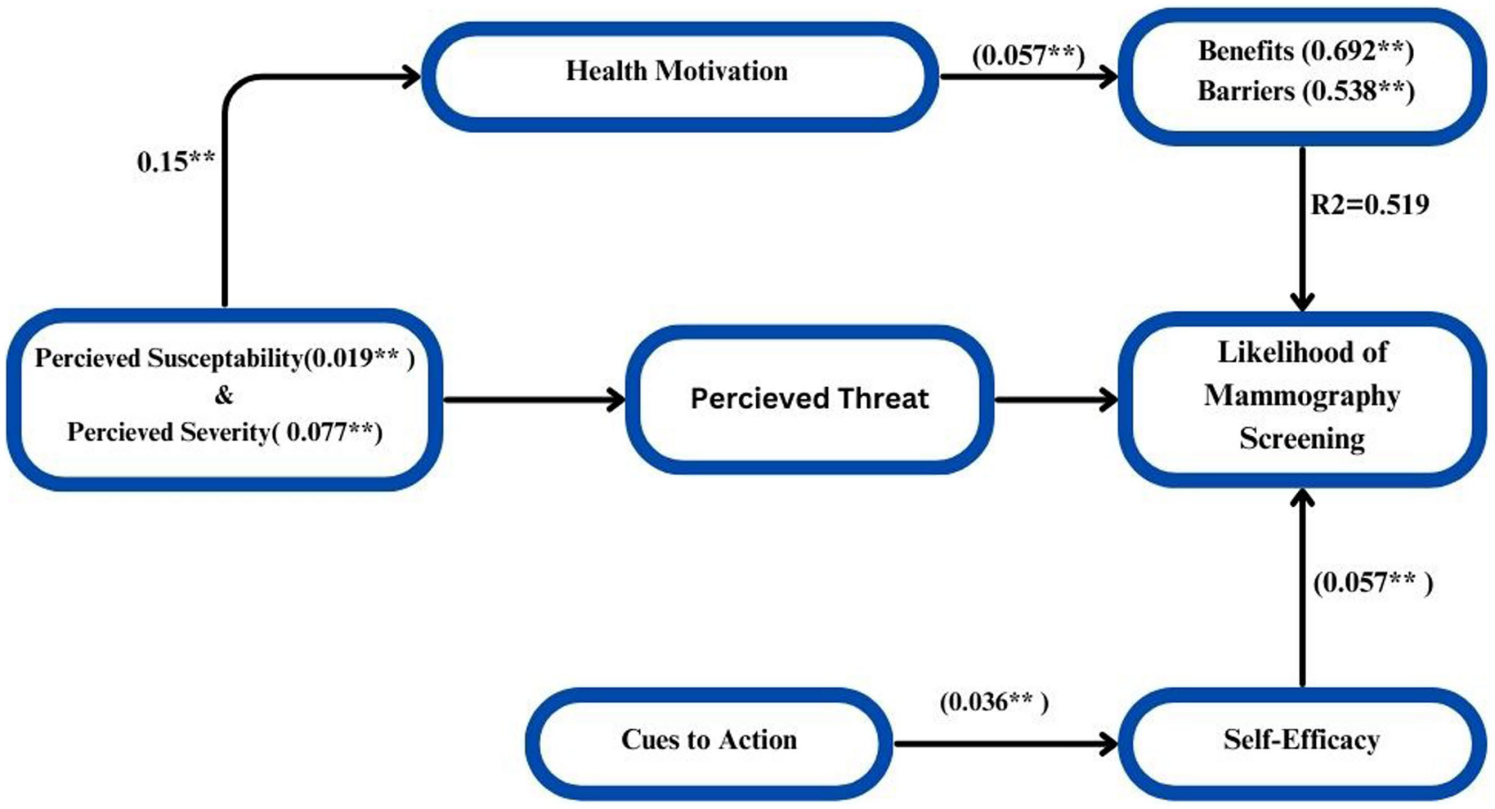
Final structure model with all HBM hypothesized causal paths. **p < 0.001.

In order to estimate the values of the coefficients in the underlying linear model and ascertain the direct and indirect effects of variables (HBM), path analysis was carried out. Measuring the direct and indirect effects of a set of independent variables on a dependent variable, path analysis is just a standardized partial regression coefficient that divides the correlation coefficients. The term “model identification” describes the number of items we must estimate (e.g., the path coefficients and correlations) in relation to the amount of information that can be derived from the data, be it about the observed variances of the variables or the covariance between them. The quantity of information in this regression model is just the number of paths that need to be estimated; it is easily identified. The result would be concluded that a causal model deleting the direct influence of threat and the indirect influence of susceptibility and severity channeled through benefits and barriers fits the data more strongly than did the model including these paths. The hypothesized causal paths effect of the model was explained by 64.3% that shows there is strong relationship of the variables significantly (*p* < 0.005). The path analysis model yielded direct and indirect effects, which are displayed in Figure 9 to illustrate the interaction. Final path model fitted for six hypothesized HBM constructs =0.019 Susceptibility + 0.077 Severity + 0.692 Benefits + 0.538 Barriers + 0.057 Self-Efficacy + 0.036 Cues to Action + 0.442 (Figure 9).

## Discussion

This randomized controlled trial was carried out using the core elements of the health belief model, followed by a qualitative study that provided insights into the design and development of health education interventions for mammography screening. Qualitative and cross-sectional studies at baseline found that there was a strong interplay between perception and breast screening (published elsewhere) (21).

Model-based interventions to improve mammography are generally effective across each construct, at least in terms of improving perceptions of screening and general knowledge. This is similar to studies conducted in several parts of the world that found that education based on tasted behavioral models increases the likelihood of adopting a certain behavior when appropriately targeted (25–27). This finding is also supported by the fact that some studies using single behavioral approaches targeting patients were ineffective, confirming those multi-approach interventions were successful (26, 28). The possible explanation could be that no study focuses on the intensity of optimal intervention where the doze and peak of the intervention are reached rather than simply giving education intervention on the specific behavior of the interest. This also suggested that there was a strong interaction between perception and mammography screening in the qualitative and baseline survey of this study, which was published elsewhere (21).

This study found that repeated health education interventions increased the knowledge of the intervention group of participants significantly and expressed a knowledge variance of 77.8% at different points in time. This figure is greater than the results obtained in various interventional and systematic reviews synthesis studies (15, 16, 25–28). This might be the current study, which used various methods to demonstrate mammography screening methods and intense mammography education every fifteen days. The present study found that after three and six months of the intervention, the intervention group had a statistically significant increase in threat appraisals (susceptibility and severity), efficacy appraisals (benefit outweighed barrier), and self-efficacy and knowledge of the study participants. This is similar to the several systematic reviews and meta-analyses conducted in the mammography use education that documented model-based education on breast screening as having a successful ending (26, 28, 29). However, naturally, the uptake of breast screening behavior varies from place to place. Our preceding baseline qualitative findings showed a strong interplay between perception and mammography screening and were published elsewhere (21).

The systematic review on health promotion interventions as a way of knowing the situation of the world and local evidence for this randomized trial showed that mammography screening is determined by the actual accessibility of the services and affordability of the individual women (28). The current study found that though the actual screening is low, the intention to have mammography screening has shown statistically higher scores of barriers and lower scores of benefits.

The current study found that health motivations and valuing one’s health status significantly explained higher variances in the intervention group than the control group. Naturally, after certain reminders, people value or are motivated to be healthy in their lives (30, 31). Previous publications as well as successful motivational interventions confirm the persuasiveness of personal and individualized risks (32, 33). This is supported by the concept of HBM, which states that the perceived benefits of individuals increased where there were no or limited barriers to hinder preventive health behavior (18, 19).

The current study also found that the practice of actual breast screening by mammography showed no change after intervention. Previously published studies also supported this idea (34–36). This is supported by the concept of HBM, which states that the perceived benefits of individuals increased where there were no or limited barriers to hinder preventive health behavior (19). The possible reason for this could be that mammography is largely situated in the center of Ethiopia, and the costs associated with it are extremely high.

This study’s strength is that it ensures and promotes more control over the intervention, allowing for a clear distinction between the intervention and control groups. Another advantage of this randomized controlled trial is that it produces an unbiased estimate of the effect for both the intervention and control groups. To my knowledge, this is one of the first randomized controlled trials in Ethiopia to apply community-based interventions, which may aid in recognizing the value of intervention rather than simply describing them.

As limitations, because this randomized controlled trial intervention is based on health belief model constructs, thus the model is a psychological model, it does not take into account other factors that may influence health behaviors, such as environmental or economic factors, social norms, and peer influences, indicating the need to fulfill enabling factors. The other limitation of the model is that it may have a gap between actual behavior and psychological responses, i.e., participants who were in a positive zone may not be in a protective zone, which may lead to over-reporting of safe behavior. The possible limitation is that the sufficiency of information may vary depending on the person delivering health education, though the intervention document is the same. The other possible limitation is a trial using health education interventions at the district level, in case information contamination may exist due to the nature of behavioral intervention research.

In conclusion, the study emphasizes the advantages of HBM interventional initiatives, such as educational and motivational programs, in improving public perception of mammography screening. Repetitive education has been shown to improve comprehension of the mammography problem and increase willingness to attend screening. Though a slight difference is normal, there was no significant difference between the districts; instead, all intervention districts had significantly higher perceptions. This study also found that community-based intervention, followed by an exploratory qualitative approach using gap analysis, had a significant impact on mammography screening rates. The results of this study showed that actual mammography use was very low. This shows there is a need to bring the services as close as the people can get and afford to avail screening services in the community. As for future prospects, it is clear that interventions aiming to improve general breast health lead to an increase in the likelihood of mammography screening among reproductive-age women in Ethiopia. Organizations involved in breast cancer prevention and control should focus on health education programs to enhance mammography use benefits and increase women’s self-efficacy in screening. Future researchers should examine the intensity and range of information to determine the optimal intervention dose and peak.

## Data Availability

The original contributions presented in the study are included in the article/supplementary material, further inquiries can be directed to the corresponding authors.
